# The 2024 Taiwanese Presidential Election on social media: Identity, policy, and affective virality

**DOI:** 10.1093/pnasnexus/pgae130

**Published:** 2024-04-05

**Authors:** Ho-Chun Herbert Chang, Yu Sunny Fang

**Affiliations:** Quantitative Social Science, Dartmouth College, Hanover, NH 03755, USA; Computer Science, Barnard College, New York, NY 10027, USA

**Keywords:** Taiwan, social media, pragmatic bias, political communication

## Abstract

The 2024 Taiwanese Presidential Election is not just a critical geopolitical event, it also engages with themes of alternative candidacy, foreign policy, and affective polarization. At one point, a four-candidate race had emerged in a traditionally bipartisan election, with alternative candidates disrupting the dichotomy of Chinese vs. Taiwanese identity. Leveraging 911,510 posts and 101,600,047 engagements on social media, we analyze user discourse and engagement. First, we find traditional candidates derive more engagement on foreign policy and geopolitical issues, alternative candidates on domestic issues. Additionally, virality is generated by affective reasons, although in-group references generate more engagement than out-group references. Lastly, a puzzle is revealed where alternative candidates draw more homogeneous attention from national identity groups. Results suggest alternative candidacy can be generated by both positive and negative comparisons rooted in national identity.

**Significance Statement**

The year 2024 is the largest election year in history, with citizens from more than 50 countries slated to vote. The 2024 Taiwanese Presidential Elections has drawn significant international attention, where in a traditionally bipartisan race, two new candidates emerged to challenge the political norm. Our analysis of 101,600,047 engagements on social media suggests how third-party candidates may emerge: through a focus on domestic policy, skepticism toward foreign allies, and both positive and negative engagement with national identity. Our work lays the groundwork for how political leaders may engage with voters in light of heightened global competition and conflict.

## Introduction

Dubbed “the world’s most dangerous place” by the Economist (1, 2), Taiwan has continued its role as a geopolitical lynchpin due to its role in mediating the US–China global competition and in producing the world’s silicon chips (3, 4). As a result, the 2024 Taiwanese Presidential Election has garnered significant international attention.

In prior Taiwanese elections, the pan-Blue alliance led by the Kuomintang (KMT) and the pan-Green alliance led by the Democratic People’s Party (DPP) embodied competing visions of the nation’s identity—Taiwan vs. the Republic of China (ROC). However, over the summer of 2023, two alternative candidates emerged: Ko Wen-Je of the Taiwan People Party (TPP) and Terry Gou, who entered as an independent. This caused significant concern to the camps of Ko, Gou, and KMT candidate Hou, who believed they were dividing their bases (we summarize each candidates’ party, notable policy stances, and affiliations in Table 1). United by their dislike for the DPP, the KMT and TPP proposed the “blue–white alliance.” The parties agreed that the poll leader between Ko and Hou would be the president. However, within three days the potential alliance collapsed, with Ko rejecting the rules of the coalition. Reports indicated that his base was unhappy with his agreement to the terms, and acceptance would alienate his supporters (5).

**Table 1.** Four viable candidates (before 2023 November 10), their affiliated party, and notable policy stances.

	Party	Notable policy stances
Lai Chingte	DPP	Exchange with China as equals, improve defense capabilities. Substitute nuclear energy by 2030.
Hou Youyi	KMT	Maintain peace under 92 consensus. Minimum wage at 33,000 NTD. Full utilization of nuclear plants.
Ko Wenje	TPP	Do not rely solely on defense. Adjust minimum wage via formula. Full utilization of nuclear plants.
Gou Taiming	Ind.	Cross-strait engagement through 92 consensus. Full electric vehicles by 2030. CEO of FoxConn & previously KMT.

How do these groups diverge? What issues do supporters of each group respond to, and how do candidates engage their supporters differently? To investigate this, we first look to the dominant identity-based cleavage in past elections—national identity. National identity, though more frequently bound by ethnic and civic commonalities, can also be shaped by a “significant other” that bears geographical proximity and is a perceived threat to the nation’s sovereignty (6). In Taiwan’s case, the “China factor” has long played a critical role in its elections, with citizens reconciling their cultural inheritance from China with their Taiwanese identity (7).

It is useful to discuss the roots of Taiwanese and Chinese identity. In 1949, Chiang Kai-shek, the leader of the Chinese Nationalist Party, or KMT, retreated to Taiwan after their defeat by the Chinese Communist Party. Although he treated Taiwan as a temporary military base to stage his reclamation of the mainland, Chiang attempted to “re-Sinicize” residents of the island, emphasizing cultural, ethnic, language, and historical ties to China to induce a Chinese identity (8). However, in the 1980s, Taiwan underwent a period of rapid economic growth and democratization. Lee Teng-hui became the first native-born to assume office in 1988 (9). Simultaneously, a sense of discordance emerged due to the lack of international recognition (8). The interplay of democratization at home and ostracization abroad produced targeted public diplomacy that enhanced Taiwan’s international recognition as a progressive liberal state in contrast to authoritarian China.

These developments engendered a new Taiwanese ethnic and national identity, and eventually the multiparty system in Taiwan. In 2000, the Democratic Progressive Party (DPP) won the presidential seat and heralded the rivalry between the pan-Blue Alliance and the pan-Green Alliance, led by the KMT and DPP, respectively (8). As a growing population renounced their Chinese identity, those identifying as Taiwanese leaned toward Taiwanese independence and the DPP, who espoused the idea (10). The dichotomy that determined prior elections could be summarized as such: pan-Blue indicates preference toward the ROC, Chinese consciousness, and pro-unification; pan-Green indicates a preference for Taiwan as a national identity or a Taiwanese consciousness (7).

The emergence of Ko and Gou suggests that this dichotomy may no longer be sufficient. Alternative candidates must fall on this axis of national identity, overlaps of ethnic identity and state identification. However, the conflation and interchangeability of the three when discussing Taiwan’s national identity are common but misleading (11). To illustrate this distinction, consider two ways to ask about identity:

- Do you consider yourself Taiwanese, Chinese, or both?
- Should we call ourselves Taiwan and the ROC?

The first elicits ethnic identity whereas the second states identification. While this distinction may be subtle, there are many cases where this mattered in decision-making. For instance, under the Lausanne Agreement with the International Olympic Committee, Taiwan’s national team has been competing in international sports events under the name “Chinese Taipei” since 1981 (12). The referendum asks, “Do you agree that ‘Taiwan’ be used as the [country’s] full name when applying for participation in international athletic competitions and the 2020 Tokyo Olympics?” (13).

Despite a majority identifying as Taiwanese in 2018 (14), the referendum failed with 24.11% of eligible voters voting in support and 29.23% voting against (15). Most believed participating in international competitions as “Taiwan” was impossible/difficult and feared retaliation from Beijing (16). Chinese Taipei was, in contrast, an acceptable compromise.

The results demonstrate pragmatic bias. Pragmatic bias, defined by Corbett et al., is the tendency to withhold support for members of groups for whom success is perceived to be difficult or impossible to achieve (17, 18). While this concerns candidate electability, this extends to the tradeoffs between enacting pragmatic foreign policy and identification in a vacuum. It would explain the paradox where although a majority identify as Taiwanese, they still shy away from calling the country Taiwan.

Beyond national identity, the appearance of Ko and Gou also asks how alternative candidates align on policy issues, especially between domestic and foreign policy (19). Gaining prominence in the December 2014 local elections, Ko has a track record of emphasizing economic and governance issues and presenting a centrist position on cross-Strait relations (20). Focused on pragmaticism, Ko and the TPP’s emphasis on domestic policy issues stood in contrast with his traditional counterparts.

Based on prior elections, we would expect foreign policy to take precedence over domestic issues. In the 2020 election, Rauchfleisch et al. found that the issue of China ranked first, followed by economic policy (21). This would advantage traditional candidates as alternative parties are characterized by their focus on domestic issues (22). Moreover, independence vs. unification frequently acts as a latent confounder that has the power to polarize issues (23). In the United States, during the final period of the Cold War, foreign policy issues in presidential elections were substantial and comparable to economic circumstances (24). With increased global tensions through the Russian invasion of Ukraine, the US–China global competition, and the Israel–Palestine conflict, the salience of foreign policy will likely extend not just to Taiwan but to elections throughout 2024.

In addition to domestic and foreign policy issues, another factor is partisan dynamics: whether support is due to actual policy issues or due to favoritism and animosity to their own party and opponents. To investigate this, we turn to social media. After the Cambridge Analytica Scandal in 2016, a significant stream of research has been dedicated to understanding how social media contributes to elections and polarization (25–28). There are two general types of polarization: attitudinal, which denotes disagreement over issues, and affective, which is due to dislike of opposing parties (29, 30). In the American context, Rathje et al. found that out-group animosity generates the most virality (31) using Twitter and Facebook posts by congress members. As such, understanding how references to partisan in-groups and out-groups, and the toxicity of language employed (as a measure of negative affect), is a crucial complement to analysis of the direct policy issues.

### Research questions

National identity, partisan identity, and policy issues are all important considerations for the 2024 Taiwanese Presidential Elections. The emergence of alternative candidates breaks down the traditional dichotomy of national identity and approaches to foreign policy. Simultaneously, alternative candidates and parties often emphasize domestic issues, bringing to bear the tension between foreign–domestic debates. Lastly, support for candidates may arise from both attitudinal and affective reasons as well.

As the two most dominant platforms in Taiwan are Facebook and Line, with diffusion rates of 89 and 85%, respectively (32), and due to Line’s closed chatroom nature, we investigate these factors using Facebook. Not only does the Facebook API provided through CrowdTangle provide the communication and engagement of the candidates and parties, but it also provides public groups, which reveal wider discussion by everyday users, and as such, are likely the best proxy for understanding public discourse. Moreover, understanding the dynamics of social media during elections is significant due to the critical question of whether it polarizes. Our study contributes to this larger body of work as well. As such, we have the following **research questions**.

- *RQ1:* How do traditional and alternative candidates, and their supporters, diverge in their engagement with foreign and domestic policy issues?
- *RQ2:* Do references to policy issues or partisan identity generate more virality?
- *RQ3:* How do traditional and alternative candidates diverge in their engagement with national identity?

## Data and methods

### Data collection

To scrape social media, we searched Facebook and Instagram using CrowdTangle, directly searching for elections-related discourse and the candidates. There was significantly more content on Facebook than on Instagram, which adheres to previous findings about the less political nature of Instagram (33) and the greater usage of Facebook in Taiwan, relative to Instagram. This served as a control and we focused our analysis on Facebook. As mentioned prior, Facebook public groups are the best proxy for understanding public discourse, since the alternative of scraping private groups and personal timelines on Facebook is rife with ethical questions of consent.

Table 2 shows the types of searches we conducted. A list of keywords is included in the Supplementary material. When scraping the official candidate and party page, we discovered the DPP had separate pages for their official spokesperson, their youth representatives, and their legislative updates. In comparison, the KMT and TPP had one official page. We then took particular care to scrape posts from public groups, which consisted of 63.9% of all the total posts. This gives a sample of the discourse. We used dates starting from 2023 December 1 to capture all content exactly a year prior to when the elections are held.

**Table 2.** List of searches on CrowdTangle.

Data Source	Description
General Elections	Searched on keywords pertaining to the presidential election (i.e. 大選).
Official Candidate and Party Pages	Searched on each candidate’s official page. This included Tsai Ing-Wen and Han Kuo-Yu due to their large presence in the overall dataset
CrowdTangle Candidate General	Search on keywords by candidate. This includes all mentions of the candidate including in oppositional groups
CrowdTangle Candidate and Identity Community	Using the Candidate General dataset, we produced a manual subset of public candidate support groups, i.e. 挺賴團 (Lai support group). Also includes groups that explicitly mention Taiwan and ROC, such as 中華民國粉絲團 (ROC Fan Club)

A full list of the groups is included in the Supplementary material. Tsai and Han were included as they were prior presidential candidates, who may garner significant referrals (34).

### Topic modeling and toxicity labeling

To gauge topic salience for our first question, we extracted keywords from datasets for General Elections and CrowdTangle Candidate and Identity Community. To do so, we utilized jieba (35), a Python Chinese word segmentation module. Since jieba was primarily trained in Simplified Chinese, the chinese-converter (36) library was used to convert the text data from Traditional Chinese to Simplified Chinese before conducting keyword extraction.

We first tagged the two datasets by three main categories: political figures (e.g. the four candidates), geopolitics (e.g. the United States), and domestic issues, which we adhered to the policy issues outlined by Sheng and Liao (37) and added relevant topics such as technological advancement, seen as Taiwan is often mentioned in the discussion of the global semiconductor race (4). After filtering the data sources by policy issues and candidates, we employed the two keyword extraction methods: frequency counts and latent Dirichlet allocation (LDA) for topic modeling. LDA works by selecting the number of clusters that yielded the largest coherence value (38). To measure toxicity, we use the Google Perspective API frequently used in political science research to operationalize incivility (39).

To visualize potential cleavages, we subset on all candidate posts pertaining to each subject and then perform BERT-embeddings. BERT is a large language model architecture that encodes textual information in a high-dimensional vector space. Using sentence-BERT, we embedded the posts into a 384-dimensional vector, took the mean by each candidate, and then projected the mean representation into 1D space using principal component analysis (PCA). Barring equality in distance, this allows us to preserve the ordinal relationship between each candidate and how they discuss policy issues.

### In-/out-group labeling

To answer our first question, we sampled Facebook posts from the support group for all 4 candidates and manually coded three different variables: *self-identification*, *group selection*, and **State identification preference**. Some heuristics used are as follows: *self-identification* is 1 if the text explicitly mentions “Taiwanese” (台灣人), (中國人, or 華人), 0 otherwise; *group selection* is 1 if the text references phrases “Taiwan” (台灣) or “Taiwanese citizen/population” (i.e. 台灣人民), or “Republic of China” (中華民國), 0 otherwise. The difference between “Taiwanese” and “Taiwanese citizen” is subtle but crucial: when referring to “Taiwanese,” the user speaks to their ethnic identity; on the other hand, “Taiwanese citizen” refers to legal citizens of the Taiwanese state as a collective, thus appealing to state selection. In Mandarin, this manifests as the difference between “person” and “citizen” (人 vs. 人民). State identification preference, denoted by the variable *State identification preference*, can take on three values: 1, 0, or 0.5. A 1 denotes Taiwan is preferred in reference to the country; a 0 means ROC is preferred. The middle case, 0.5 is rare but became necessary in our coding as candidates would attempt to appeal to both even at the group level (i.e. ROC, Taiwan 中華民國台灣).

We then coded for partisan in-/out-group messages to examine their effects on virality. The criteria for in-group used is a reference to one’s own party, themselves, and a previous presidential candidate of their own party. For instance, for Lai, this would include Lai, Tsai, and the DPP. For out-group labeling, this is the full set of candidates and parties minus their own party and prior candidate. For instance, Ko would have Tsai, Lai, Han, Gou, Hou, the DPP, and the KMT (all except himself and the TPP). The only caveat to this labeling strategy is for Hou and Gou, who used to belong to the same party. They are considered oppositional, and eventually Gou attempted to run as an independent. As political ads can be generalized as “attack,” “promote,” and “contrast,” assuming a similar intention but with a different political communication modality, this labeling process allows us to contrast these three intentions (40). We also confirm out-group references are more toxic than in-group tweets in the Supplementary material.

## Results

### Alternative candidates are moderate in issue-framing

**Fig. 1.**
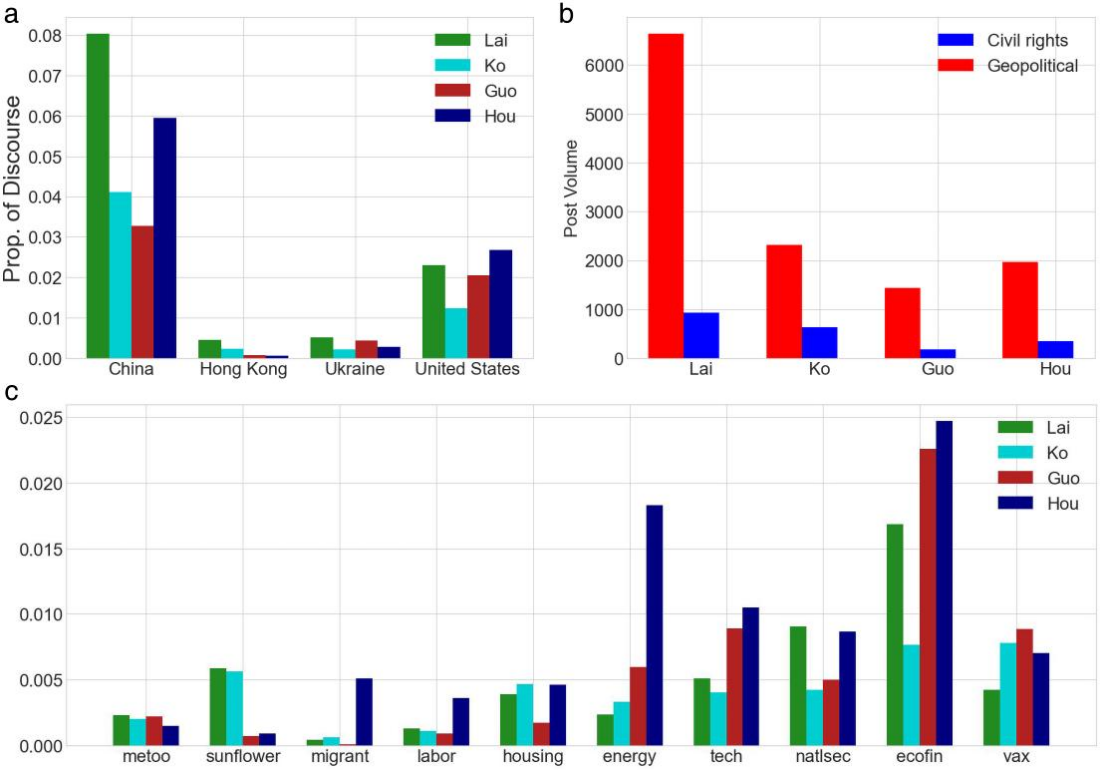
Volume of key topics discussed. a) Geopolitical issue proportion cross-sectioned with candidates. b) Civil rights vs. geopolitical topic volume by candidate. c) Domestic policy issue discourse volume.

For this analysis, we use the posts from candidate-specific communities, to better understand which topics online supporters for each candidate discuss. Figure 1a shows the frequency of geopolitical issues in these support groups, and we find Lai and Hou groups have a higher proportion of discourse regarding China and the United States. On the other hand, Lai and Ko groups reference Hong Kong the most, which aligns with their pan-Green identity. In contrast, the support groups of Ko and Gou discuss these issues at a lower frequency.

Figure 1b shows the frequency of geopolitical issues vs. civil rights—*#MeToo, the Sunflower Movement, migrant workers, labor,* and *housing.* For the support groups for each candidate, the number of posts concerning geopolitics greatly outnumber civil rights. In other words, even for the supporters of alternative candidates, geopolitical issues tend to dominate the discussion.

Figure 1c shows the posts per domestic issue normalized over the total volume per candidate group. In these supporter groups, we find traditional candidates Lai and Hou groups having a greater level of discourse regarding China and the United States, pan-Green candidates Lai and Ko groups referencing Hong Kong, and, on the flip side, pan-Blue supporters fixate more on *energy*, *tech*, and the *general economy*. Hou’s supporters asymmetrically discuss migrant workers and energy. When discussing national security, the support groups for traditional candidates (Lai and Hou) tend to be more active. Lai’s supporters also shy away from discussing vaccines. Ko, known for his emphasis on domestic policies, discusses housing more compared to the other candidates.

**Fig. 2.**
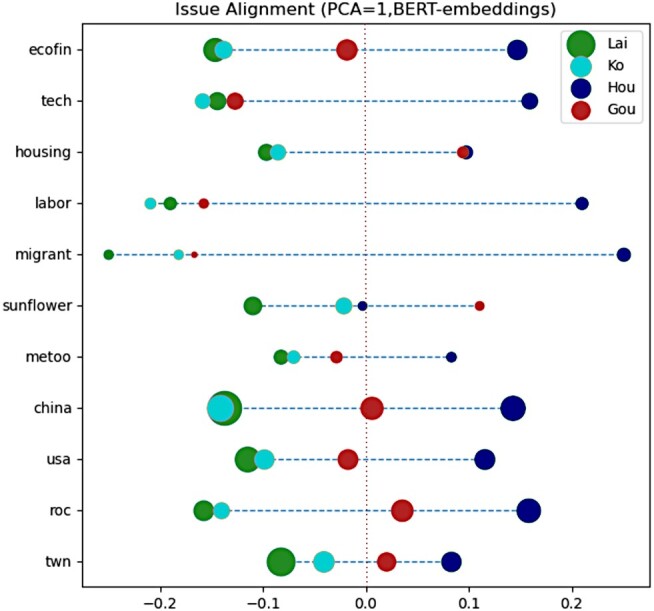
Embeddings by topic and candidate. Distance between points indicates a difference in how issues are framed by candidates.

Like the trend in Hong Kong, the Sunflower Movement is evoked in Lai and Ko support groups but rarely with Gou and Hou. This is quite curious as the “blue–white” alliance would imply supporters of Ko align with the pan-Blue camp. To further understand how candidates themselves discuss these issues differently, Figure 2 shows the average projected BERT-embeddings of each policy issue by candidate. To briefly recap, each candidate post is embedded in multidimensional Euclidean space using BERT. These coordinates are then averaged, by policy and by candidate, then projected into 1D space using PCA. As such, we can interpret each average as the candidate’s average “position” on a topic, and interpret the distance between candidates as how similarly they talk about certain issues. While this does not directly capture their stance, it overlaps and covers the context in which candidates talk about domestic and foreign issues.

In general, the two traditional candidates Lai (green) and Hou (blue) occupy the two poles. Each topic is centered around 0 and the two poles, to show where the two other candidates lie. Curiously, Ko (cyan) frequently aligns with Lai, whereas Hou and Gou occupy the right. In other words, while Ko is frequently framed as part of the pan-Blue alliance, the way he discusses policy issues aligns more closely with Lai. Additionally, there are policy issues for which Ko occupies the poles, such as tech and labor.

In considering what candidates say and what their supporters discuss, we answer RQ1. Geopolitical issues concerning national security are emphasized by supporters of traditional candidates; Taiwan-based identity issues like the Sunflower Movement are emphasized by pan-Green supporters. Macroeconomic policies like energy, tech, and the economy are emphasized by pan-Blue supporters. Overall, alternative candidates do tend toward the center in issue alignment. Likely due to their party history, traditional candidates focus more on foreign policy, and alternative candidates on domestic policy issues.

### What drives virality?

Having investigated the topical overlap of both supporters and candidates, we now consider how online users **engage** with candidates. To be clear, this regression also contains the public party pages the candidates are affiliated with. To investigate this, we regress total interactions, love reactions, and angry reactions, on the salient topics and group status. Our strategy is to understand the average effect for the presidential candidates and parties overall, then also by each individual candidate. As such, we build a pooled ordinary least-squares regression (OLS) for the first, and then individual OLS models for the latter.

**Fig. 3.**
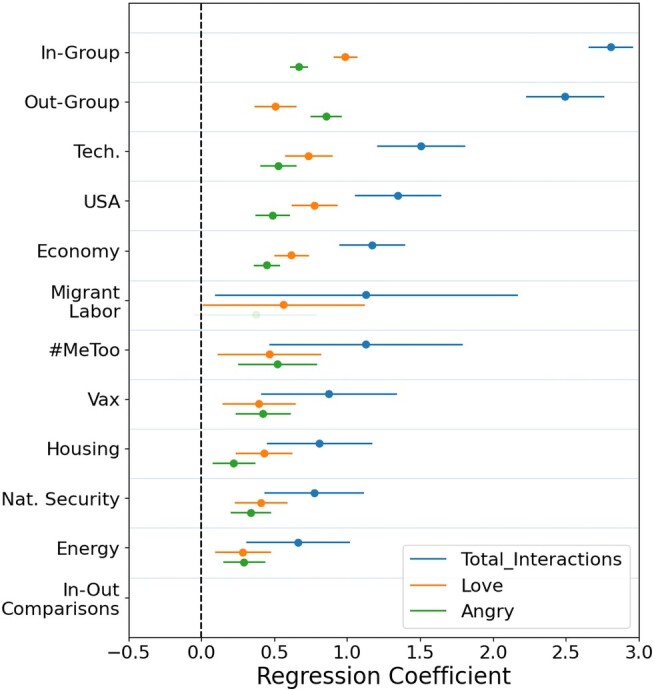
Regression results for total interactions (blue), love reactions (orange), and angry reactions (green).

Figure 3 shows the overall regression results for policy issue-based indicator variables and in-/out-group labeling, based on total reactions (blue), love reactions (orange), and angry reactions (green). Points denote coefficients and the horizontal line is the 95% CI. We only show variables that are significant across the three engagement categories. Hence, Hong Kong, Ukraine, and China are not mentioned, though they are relevant to other categories.

For all three dependent variables, policy reference to the United States, MeToo, migrant workers, housing, energy, tech, national security, the economy, and vaccines all produce positive effects (except for migrant workers on angry reactions). What is curious is the absence of China. One theory is that this is due to its ubiquity in election discourse, and thus makes no statistical increase in reactions. In contrast, foreign policy regarding the United States has a much larger impact, shadowed only by reference to Tech. These two topics often go hand-in-hand, due to the core theme of Silicon Diplomacy and debates on TSMC in Arizona. Together, these point toward the increased role of foreign relations with the United States in contrast to previous election cycles; we discuss its implications for other countries further in the discussion.

More importantly, group labels tend to produce the greatest effect, where in-group mentions generate a 2.81 coefficient (645 interaction increase) and out-group labels produce a 2.49 coefficient (309 interaction increase). These results stand in contrast to the findings of Rathje et al., where out-group mentions appear to be a larger driver of overall engagement (though they consider a much larger set of Congressional Members from the United States). As a test, we find in-group mentions drive the love reactions, then out-group mentions drive angry reactions, respectively, as the orange and green points swap places for in-group and out-group mentions. Additionally, when both in-and-out groups are mentioned, diffusion actually decreases, which shows direct comparisons in the same post may be detrimental to diffusion (numerical values are given in the Supplementary material, along with group cross-sections with toxicity to show out-group mentions are more toxic).

**Fig. 4.**
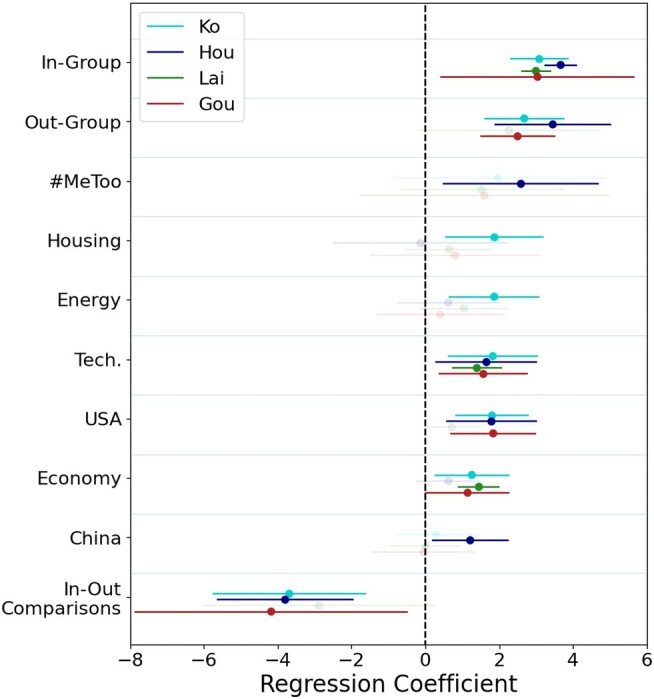
Regression results for the three primary engagement metrics.

Next, we conduct the same regression for each of the four candidates individually, shown in Fig. 4. Once more, we find in-group mentions are crucial to driving diffusion. There are a few trends to note. First, Hou’s engagement is the only one that elicits a reaction in mentioning China. Although this may seem strange, one explanation is that China is such a ubiquitous topic of comparison, that it does not generate substantially more diffusion among the communities of other candidates. Other issues such as the United States, defense, and technology may all reference China—and hence act as an invisible confounder. What is more likely is Hou’s economic discourse is inherently tied to China. When observing what is statistically significant, Hou is also missing in significance for the general economy, because his economic discourse is tied heavily to cross-strait relations. Curiously, Lai’s recipe for total engagement is simple, with just three indicators generating statistically significant results (in-group mentions, the overall economy, and technology). The absence of out-group mentions and in-/out-group comparisons suggests his supporter base may be less sensitive to out-group animosity.

Other important trends include the extent to which alternative candidates, especially Ko, generate engagement by discussing domestic policy issues. In fact, only one of the 10 factors—China—shown is insignificant for Ko. This aligns with prior work that describes an alternative candidate’s base as a greater portion of domestic issues.

**Fig. 5.**
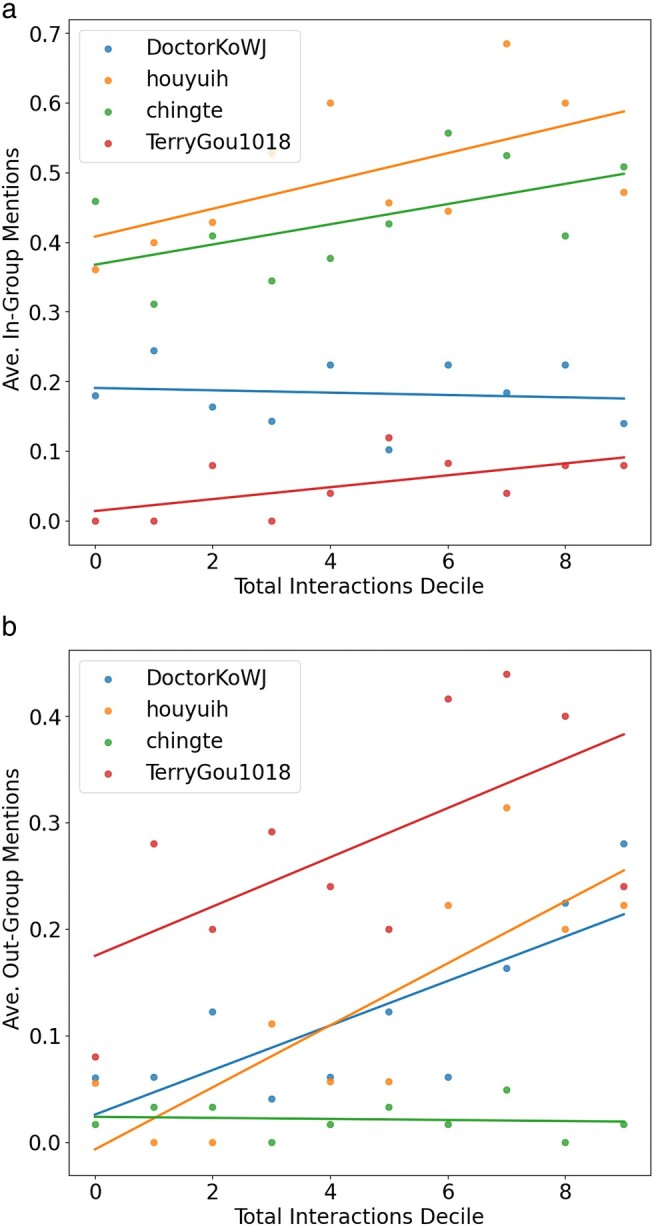
Regressions by candidate, by interaction decile against a) in-group mentions and b) out-group mentions.

We explore the effects of group dynamics further. Figure 5a shows the effects of mentioning the in-group on virality. Both traditional candidates Lai and Hou generate an increase in interactions as in-group mentions increase. This is likely due to playing to their existing voter base and party legacy. Gou has overall fewer in-group mentions but also generates an increase in interaction. However, for Ko, an increase in in-group mentions causes a slight decrease in total interactions. This suggests Ko generates virality by making out-group comparisons. Figure 5b shows the effects of mentioning the out-group on virality. Here, Gou has the most out-group mentions, whereas Ko and Hou are roughly the same. Virality increases for Hou, Ko, and Gou as out-group mentions increase. However, Lai’s virality remains agnostic to out-group mentions.

In sum, references to partisan identity generate more engagement overall, compared to any individual domestic or foreign policy issue. However, in-group mentions tend to drive engagement. Alternative candidates will generate more virality from domestic issues, and a corollary is that they may rely on more out-group comparisons to drive their stance home. This helps answer RQ2.

### Alternative candidates are less diverse in engagement

Having investigated what candidates say, what supporters discuss, and how they interact, we identify a few interesting trends. While there are concerns alternative candidates like Ko could split voter bases, his online discourse seems more aligned with Lai and responds to talking points of the pan-Green alliance like the Sunflower movement and Hong Kong. To understand this, we turn our attention to another axis of identity beyond partisanship: national identity.

**Fig. 6.**
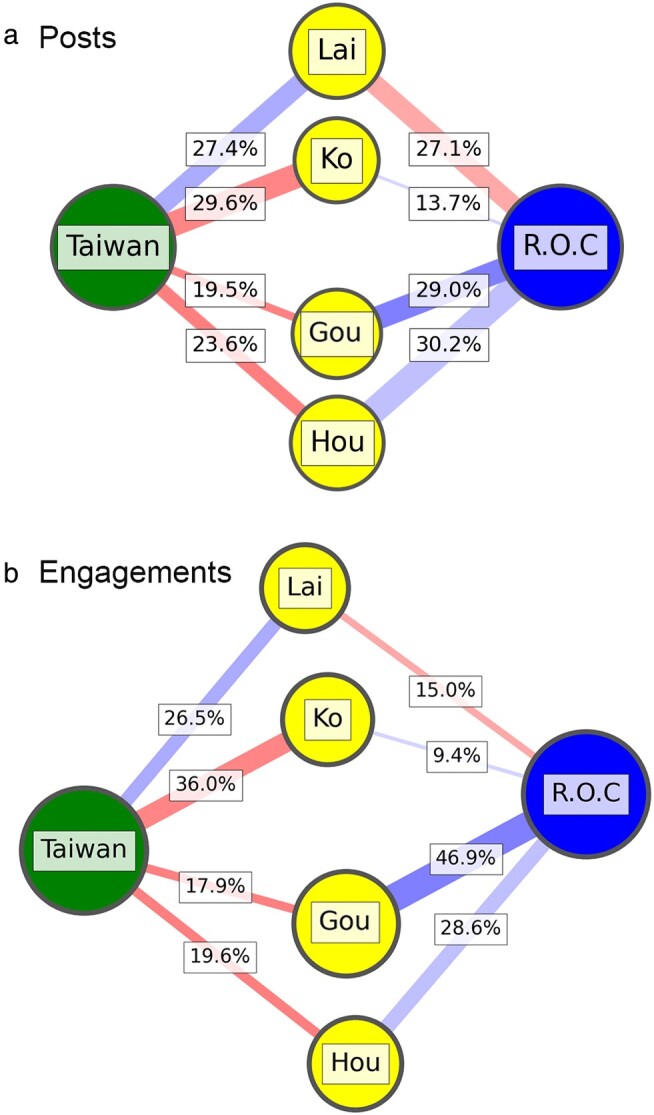
Groups include ones that reference “Taiwan” (green) and “ROC” (blue) in their name connected to presidential candidates (yellow), summarized by a) the number of posts and b) weighted by total engagements. Edge width is the number of posts about a candidate normalized over all posts in each group set.

Figure 6 shows the network of Taiwan-affiliated public groups (green), ROC-affiliated public groups (blue), and the four candidates (yellow). The edge weights are the proportion of mentions of each candidate, by the public groups (vertically they sum to 1). The colors are the favorability toward that candidate based on Eq. 1:


(1) $$\text{Fav}(\text{Cand},\text{Groups})=\frac{\left|\{\text{Love + Care}\}\right|}{\left|\{\text{Love + Care}\}|+|\{\text{Angry}\}\right|}$$

The number of love and care reactions are normalized over the sum of the two and the number of angry reactions, which are shown from low (red) to high (blue) in the network edges of Fig. 6. A full visualization with all groups is included in the Supplementary material.

There are a few immediate observations. First, for posts in Fig. 6a traditional candidates tend to attract more diverse engagement across groups. For instance, Lai is mentioned 27.1% of the time in ROC groups and 27.4% in Taiwan groups. On the other hand, Ko and Hou have a bias in mentions in each group—Ko is mentioned 29.6% in Taiwan groups vs. 13.7% in ROC groups; Gou is mentioned 29.0% in ROC groups but only 19.5% in Taiwan groups.

Ko is curious in that he captured the most attention among Taiwan groups but is negatively valenced, while is positively received in ROC groups but rarely mentioned. His large presence in Taiwan-based groups is due to direct comparisons and attacks on Ko. Translated examples include “Ko is arrogant and a liar”; less negatively-valenced include “I’m a small street vendor and I initially wanted to vote for Ko. But while he rise was built on protesting ECFA, he now supports cross-strait economic relations and I think this is dangerous.” ECFA stands for “Economic Cooperation Framework Agreement,” a cross-strait economic deal that spurred significant protest and led to the Sunflower Movement. There are two general themes. First, users tend to reference the “young people of today” being deceived by Ko, which reflects his pivot toward the pan-Blue alliance. Many from the pan-Green camp see this as a betrayal and pivot toward closer ties with the KMT.

The favorability analysis shows Lai is positively received in the Taiwan groups, whereas negatively received in the ROC groups. The dynamic is flipped for Ko, Gou, and Hou, where they are positively received in ROC groups and negatively received in Taiwan groups. Examples include support for Gou, including polls that show him ahead of Hou and Lai. In other words, there seems to be stronger support for Gou in ROC groups than even Hou. This dynamic is further exacerbated in Fig. 6b where the percentages reported are weighted by the total number of engagements. Here, Gou attracts 46.9% overwhelmingly positive engagement from ROC groups compared to 28.6% from Hou; Ko attracts 36% overwhelmingly negative engagement from Taiwan groups compared to Lai’s 26.5%.

**Fig. 7.**
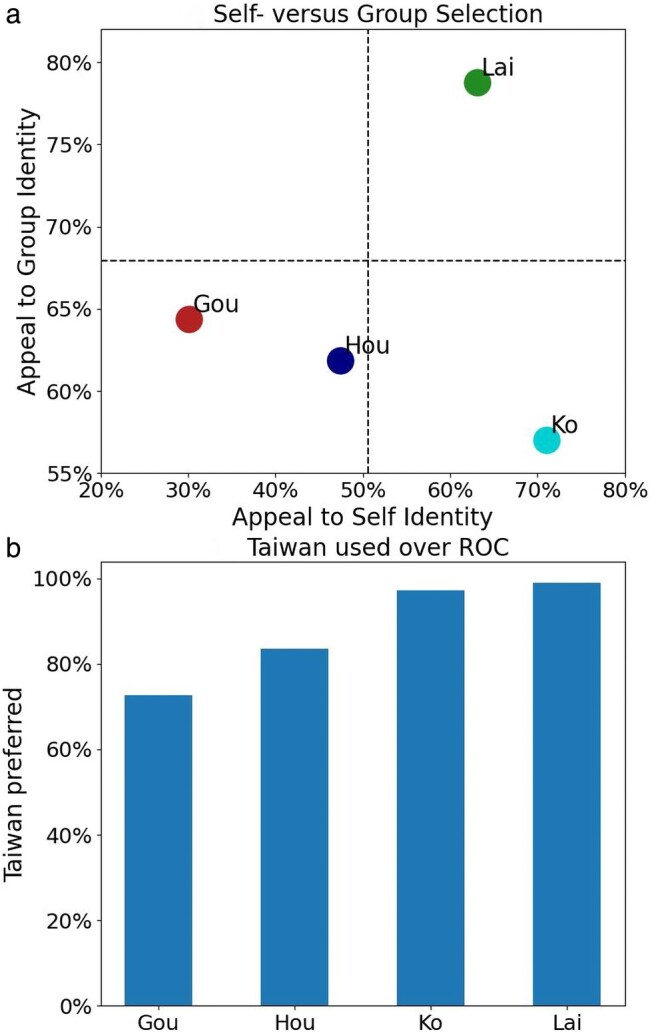
Manually coded results for a) candidate appealing to self-identification or group selection and b) preference of using Taiwan over the ROC.

A puzzle thus emerges: national identity seems to directly play a role in which candidates users of each group engage with. Alternative candidates tend to draw more homogeneous attention, whereas Ko receives little attention from the ROC groups and sharp criticism from Taiwan groups. To investigate this, we turn to the posts found in the support group of candidates. Figure 7a shows manually coded results for posts in these public groups, based on whether they appealed to self-identification (i.e. Taiwanese or Chinese) or group selection (Taiwan or ROC) in their post.

Lai’s supporters score the highest across group-identity, whereas Ko’s supporters scores the lowest. In other words, Ko’s supporters tend to frame discussion of national identity under self-identification and actively shies away from engaging with state frames. This is consistent with what we find in Fig. 6, where Ko’s support does not seem to arise from groups that are national identity-based. Note, Lai’s support groups score highly in both, and still frames discussion using ethnic identity more frequently than pan-Blue support groups. On the other hand, Gou’s supporters score higher than Hou’s for group selection but much lower in self-identification. In other words, Gou’s supporters engage more with state identity.

However, candidates themselves still prefer to refer to the state as Taiwan. Figure 7b shows the preference of candidates to refer to the island nation as “Taiwan” or “Republic of China.” For all candidates, there is an overwhelming preference to use Taiwan, especially for Ko and Lai. Interestingly, we noticed both Hou and Gou attempted to “fuse” the two terms at the group level by saying ROC, Taiwan (中華民國台灣) to appeal to those who identify more strongly with Chinese identity.

Together, this shows that alternative candidacy can be viewed through an additional axis than the existing dichotomy of state-based national identity. The explicit absence of support for Ko from these groups shows candidacy rooted in self-identity. On the other hand, alternative candidates like Gou can also emerge because he appeals even stronger toward the state selection. Indeed, Gou tends to refer to the state as Taiwan more frequently than Hou. In sum, it is possible for national identity to draw positive comparisons with traditional candidates as with Gou and Hou, and also negative comparisons as with Ko and Lai. The asymmetry of attention and engagement by alternative candidates then correspond to their relative rates of appealing to state identity. This answer RQ3.

## Discussion

With geopolitical tensions running high—from the US–China competition, the Israel–Palestine conflict, and the Russian invasion of Ukraine—foreign relations in elections may impact more than just Taiwan, but the numerous countries holding elections around the world. This case study on Taiwan shows the distinct pathways alternative candidates may take to gain relevancy, and the role affective and attitudinal factors play.

First, supporters of traditional candidates tend to discuss geopolitical issues. Traditional candidates Lai and Hou most frequently frame issues the furthest from each other, although Ko occasionally frames domestic issues like labor further than Lai. Regarding virality, references to partisan identity tend to generate significantly more engagement than both domestic and foreign policy issues. However, diverging from the US Congressional case by Rathje et al., we find in-group references tend to increase virality more for traditional candidates. On the other hand, alternative candidates often need out-group references to draw comparisons with traditional candidates. Ko’s mention of a suite of domestic policy issues is also significant in generating virality. Interestingly, China does not predict strong virality. Although it is tempting to say this diverges from prior elections, what we observe is the salience of the China factor is replaced instead by references to the United States. This indicates a shift in how the Taiwanese perceive themselves within geopolitical debates.

These results align with the largest survey (n=15,000) conducted by Commonwealth Magazine (41). The survey reports decreasing interest in the 92 Consensus and cross-strait relationships, and an increase in foreign relations. From our regression results, Taiwan’s relationship with the United States stands as one of the most crucial issues and China does not influence engagement. Although the focus has shifted to international relations and security commitments, this does not make cross-strait relations any less important. Indeed, when observing the overall volume of attention, geopolitical issues greatly outnumber domestic issues. In general, both the survey and our study find cross-strait geopolitical tensions are recast as an international one. Only Hou’s virality is positively correlated with China, which can be interpreted as KMT supporters caring more about cross-strait economic development than the DPP and TPP.

The greater effect of in-group references indicates user engagement depends more upon a candidate’s platform than dislike of the incumbent party. As our data were extracted and analyzed prior to the formation and rapid disintegration of the “blue–white alliance,” our results provide evidence of alternative candidates shying away from coalitions, in that they do not dislike the incumbent enough to reconcile misalignment of values.

However, despite the overlap of Ko’s and Lai’s supporters—such as engaging in pan-Green topics like the Sunflower movement—we find significant differences in their framing of national identity. Ko appeals considerably less to the state level whereas Lai does significantly. Moreover, Ko elicits little attention from ROC groups and negative attention from Taiwan-based groups. Alternative candidates draw more homogenous attention, and Gou in particular draws significantly more attention than Hou in ROC groups. Overall, divergences between the state and individual-level of Taiwanese identity may generate both positive comparisons (Gou and Hou) and also negative comparisons (Ko and Lai)for alternative candidates.

Although tendencies toward domestic policy issues and civic nationalism were originally championed during the Sunflower Movement in 2014, the DPP cannot rid their association with geopolitics. Just as much as KMT’s original identity is based on ethnic nationalism, so too was the DPP’s reactionary roots and the appeal to the Taiwanese state. Appeals to group selection, rather than self-identification, make this evident, and what distinguishes Lai from Ko. In posts about Ko from Taiwan-based groups, users tend to reference the “young people of today” being deceived by Ko. This is somewhat ironic as the Sunflower movement and Taiwanese independence movement have traditionally been attributed to young people, especially college students. However, with Ko’s young Taiwanese base, there seems to be a shift away from nationalism to pragmatics—both in terms of domestic policies and foreign affairs. In sum, the pathways are to either frame pragmatic and domestic concerns against established nationalistic tendencies like Ko or to double down on existing state dichotomies like Gou.

This study has a few weaknesses. First, since this is empirical, complementary surveys could help identify where political cleavages occur. Given the convergence of opinion toward the status quo, there are different opinions on how to maintain it. Maintaining the status quo should not be seen as a middle option between unification and independence, but a complex relationship between ethnic, state, and pragmatic concerns. Second, since our investigation is primarily textual, we may have missed political communication that is based on graphics. Further inquiry using vision-based multimodal models, such as visualBERT (42), would be useful to validate these results. Additionally, this study only covers Facebook. Discourse on platforms such as Line, Taiwan’s most popular chat app, and online forums such as Professional Technology Temple (PTT) and Dcard would broaden the coverage and generalizability of this study.

With the largest election year looming ahead and the majority of people in the world slated to vote, this case study on Taiwan serves as a template to understand how alternative candidates will gain traction, the role pragmaticism in both domestic and foreign affairs, and how these issues diffuse online.

## Supplementary Material

pgae130_Supplementary_Data

## Data Availability

The data used in this paper can be accessed via Meta Platform’s CrowdTangle interface: https://www.crowdtangle.com/. All methods for querying social media posts and replication are shared in the Data and methods section and Supplementary material.

## Preprints

This manuscript was posted on a preprint: https://osf.io/preprints/4t5ws.
