# Supplementary material for: The 2024 Taiwanese Presidential Election on social media: Identity, policy, and affective virality
Source: PNAS Nexus. 2024 Apr 5;3(4):pgae130. doi: 10.1093/pnasnexus/pgae130 (PMC11018540; doi:10.1093/pnasnexus/pgae130)
Supplement: pgae130_Supplementary_Data [file pgae130_supplementary_data.pdf]

Supporting Information for

The 2024 Taiwanese Presidential Election on Social Media: Identity, Policy, and Affective Virality

Ho-Chun Herbert Chang and Yu Sunny Fang

Corresponding Author name.
E-mail: herbert.chang@dartmouth.edu

This PDF file includes:

Figs. S1 to S4
Tables S1 to S6

Supplementary Materials

Love React				
	Ko (R^2 = 0.387)	Hou (R^2 = 0.597)	Lai (R^2 = 0.521)	Gou (R^2 = 0.455)
china		0.5536*		
usa	0.8774**	0.6477*		0.9562**
metoo				
housing	1.025**			
energy	0.9364**			
tech	0.8973**	0.8129*	0.5975***	
ecofin	0.5456**		0.6186***	0.7903*
in_group	1.5864***	1.622***	1.4018***	1.4619*
out_group	1.1861***	1.3636***		1.196***
in_and_out	-1.8332***	-1.711***		-2.0372*

Table S1. Regression results against the love reaction. Aligns with the results for total interactions.

Figure S2 shows the number of references made by the top seven media outlets in the dataset—Liberty Times (自由時報), United Daily News(聯合報), China Times(中時新聞), CTI News(中天新聞), Formosa TV(民視), San-Li Media(三立電視), and TVBS. These outlets also provide a balanced comparison: United Daily News, China Times, and CTI News are pan-Blue biased; Liberty Times, Formosa TV, and San-Li media are pan-Green biased. TVBS is considered centered (**?**). We find Hou received the most engagement, followed by Lai. Table S2 summarizes these values and shows why this might be the case. Immediately, CTI News features Hou at 45.8% and Gou at only 8.7%. CTI News is known for having ties with the KMT party, which may explain why they favor the traditional candidate. On the other hand, similarly blue-leaning United Daily News has a balance of coverage between Hou and Gou. The other statistic to note is that Ko is mentioned the least by the traditional media. Although this may be deliberate, a more likely reason is that when responding to ratings, the type of comparisons that generate attention diverges based on the candidate and their background. As we will see in Section **??**, candidate responses to cross-party comparisons diverge greatly. Another possibility is that the traditional media also has a steadier diet amongst the older generation, which would imply Ko would not generate as much traction.

	**CTI News**	**China Times**	**United Daily News**	**Liberty Times**	**San-Li TV**	**Formosa TV**	**TVBS**	**Average**
**Gou**	8.7%	11.0%	28.8%	21.8%	24.0%	22.9%	27.5%	20.7%
**Ko**	15.4%	17.1%	15.6%	22.3%	20.4%	18.2%	19.9%	18.4%
**Lai**	30.1%	35.7%	26.1%	26.4%	24.6%	31.8%	22.5%	28.2%
**Hou**	45.8%	36.2%	29.5%	29.5%	31.0%	27.0%	30.1%	32.7%

Table S2. Proportion of attention per candidate, normalized by news media.

Overall, traditional candidates are discussed more by the traditional media. Certainly, this may be a factor of Lai and Hou being in the race longer. However, these results were extracted using the same direct query based on candidate name and time frame, which indicates their overall greater presence in the traditional media. Inadvertently, the institutional media may exacerbate these comparisons to their core audiences.

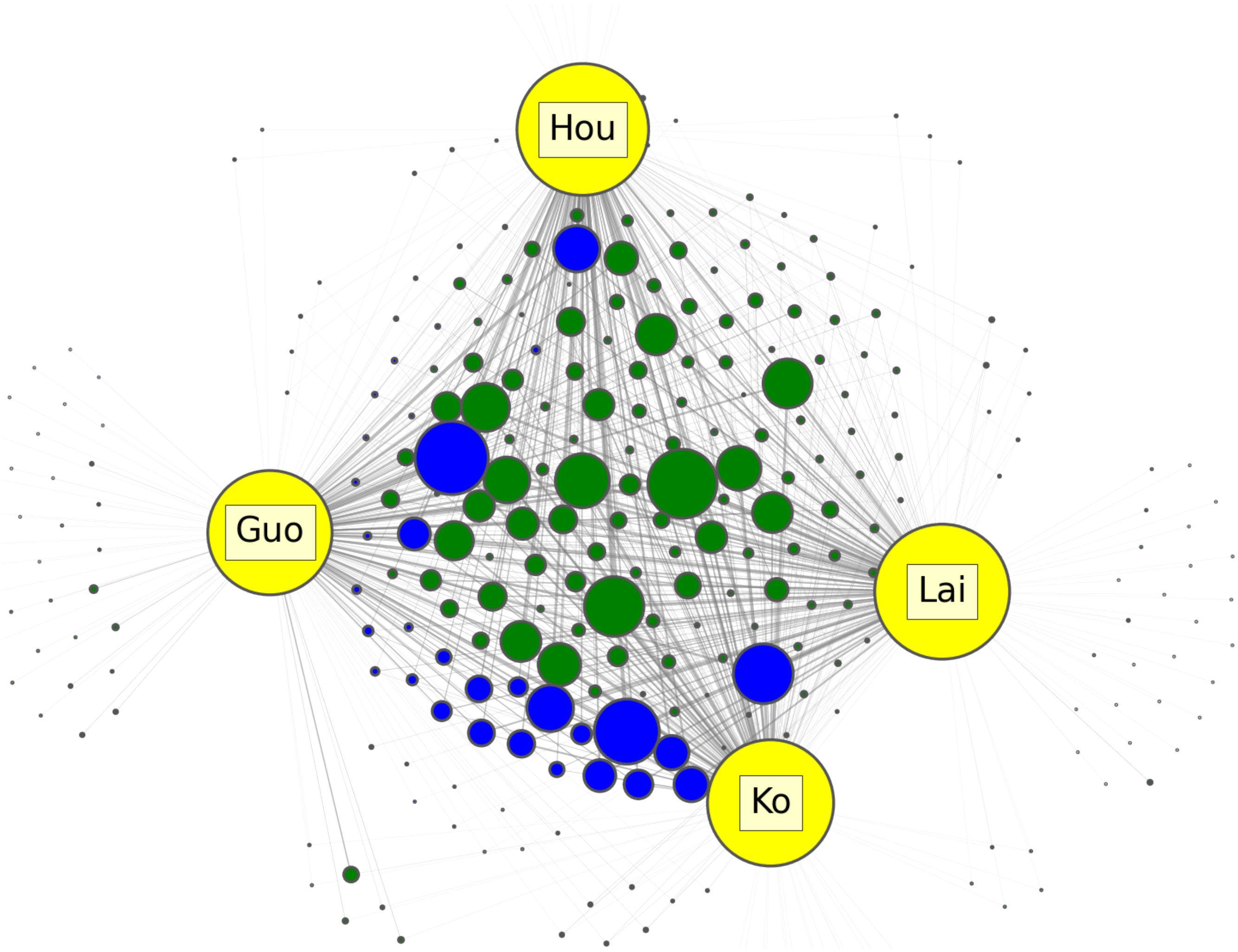

Fig. S1. Network of presidential candidates (yellow), public groups containing Taiwan (green), and public groups containing R.O.C (blue).

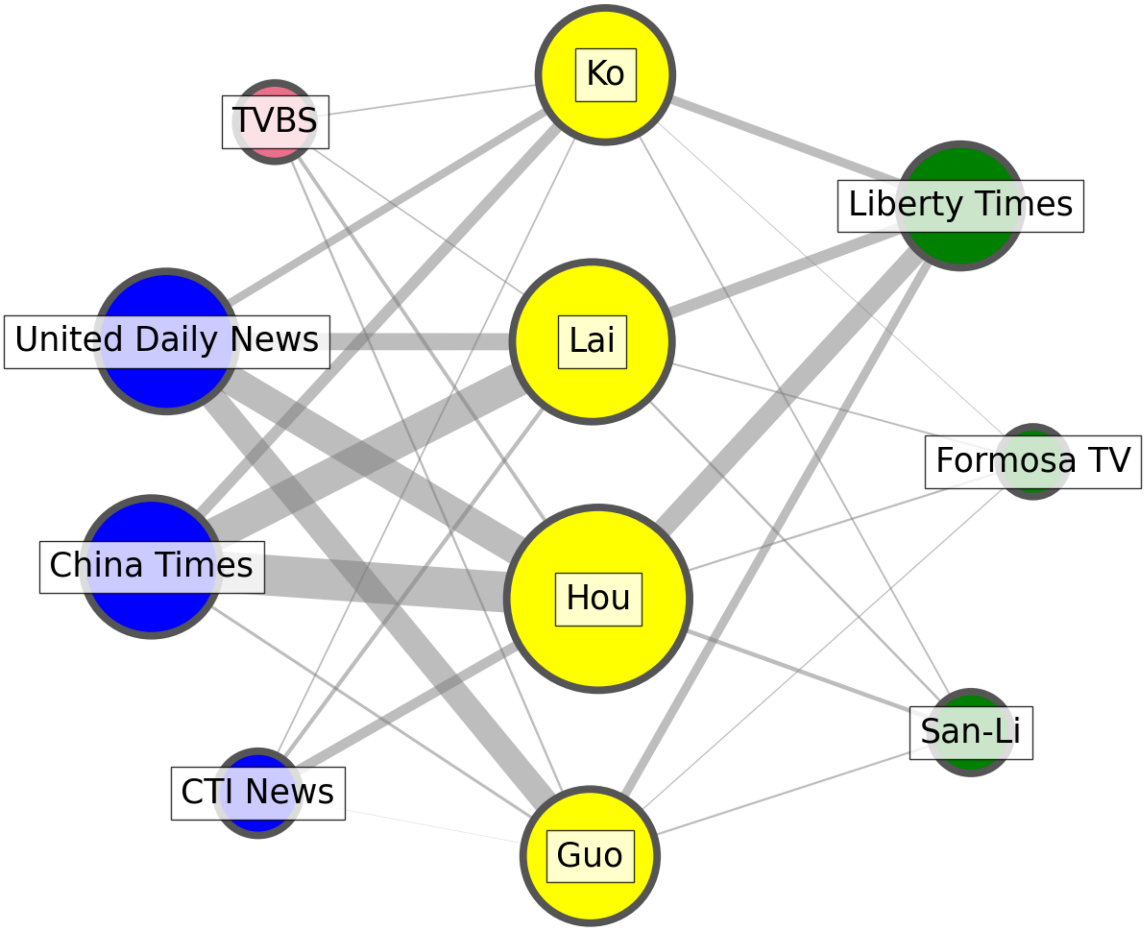

Fig. S2. Network of candidate mentioning/resharing and traditional news media outlets.

Group Name	Support	Message	Self	Group	TWN_over_ROC
郭台銘曾馨瑩粉絲團 Gou Tai-ming Tseng Hsin-Ying Fan Club	Gou	[2023“臺獨總統”誰拿了”美國CIA”准許”入門票”?還是沒拿??? 2023 <”美國CIA.AIT”臺獨戰略/授證”臺獨總統”入門票>是否已”密令指派”(一位:“國民黨KMT”臺灣總統參選人”)? 是否使得”朱立倫”KMT主席除了《徵召”非民主私下指派”》別無選擇]??? [1988若沒有<”美國CIA”臺獨戰略/密謀授證”臺獨總統”入門票>; 何來《臺灣”日裔執政”<三位”日裔”總統:”李登輝,陳水扁,蔡英文”>於1988-2000-2023》??? 2023”台灣政局”《<“美國CIA”與”日本臺獨FAPA”/獨佔臺灣”中華漢人血脈土地”政權>是否聯合幕後操控(台灣政局”台獨”戰略): “美國CIA”掌控”台灣政局”所有一切???》 於1945-2023是否從未停止]??? [19880113蔣經國死於美國CIA家法？ 46405天下雜談，30年歷史解封？太恐怖了！ 1984<老美CIA/祭出”家法”>: 壓迫(蔣經國/“美CIA”台灣副站長)用(台籍李登輝)為副手， 1988並在適當機會毒死蔣經國、 如此政權交給本土人士！ CIA做的天衣無縫， 而宋心濂因知道整個內幕， 所以死在陽明山泡澡中！ Translation: 2023 Taiwanese-independence president obtained an American CIA ticket? Or not? The American CIA AIT Taiwanese-independence strategy leaves KMT Eric Chu no choice? Did Chiang Kai Shek die to the CIA?...	1	1	0
郭台銘曾馨瑩後援會公益虎軍 Gou Tai-ming Tseng Hsin-Ying Fan Club Support Group Public Tiger Army	Gou	郭董説的對 兩岸要和 台灣更要强 超超超期待郭董下個月要給台灣人民的强國之策！ #沒有勇氣與承擔只會選舉宣傳語言企圖得到一時聲量胡言亂語的人通通沒有資格當政治領袖 #2024中華民國派一起把台灣阿銘送進總統府 Translation: Gou is right, for peace across the strait Taiwan must be strong. I'm very very very excited for Gou's strategy next month to strengthen the country. #PeopleThatSay-GarbageHaveNoRightToBeALeader #2024 supporters of the Republic of China should send Gou into the President's house.	1	1	0.5
侯友宜競選總統粉絲團 Hou You-Yi Presidential Election Fan Club	Hou	華府學者追問「九二共識」 侯友宜：回歸憲法還原真實内涵 國民黨總統參選人侯友宜今（18）日上午前往華府布魯金斯研究院會談，據了解，美方聚焦【台美關係】、【兩岸關係】、【國防自主】和【能源政策】四大面向。 面對未來若當選總統後如何處理兩岸關係，以及對於「九二共識」看法。 侯友宜明確回應，遵守【中華民國憲法之下的九二共識】，誓言保衛台灣、展現台灣自我防衛決心。 Translation: In response to scholars from DC asking about the 92 consensus, Hou response:... he will abide by the Republic of China's interpretation of the 92 consensus, and promises to protect Taiwan and defend Taiwan with determination.	0	1	0.5

Table S3. Example Messages for Self-Identification, Group Selection, and Preference between Taiwan and ROC (truncated)

Given the salience of geopolitical issues, figure S3 further cross-sections the four geopolitical issues. Note, we forgo ”Likes” as they account for around 90% of all interactions. China and the USA elicit high levels of "Haha” reactions, whereas Hong Kong and Ukraine elicit "Love”, "Angry”, and "Care” reactions—a combination of solidarity and outrage. The invasion of Ukraine in particular elicits a significant number of ”Sad” reactions.

Figure S4a) shows the toxicity scores for all candidates, where we find the KMT and TPP with the highest scores, then Gou, Ko, Hou, and Lai in order. In general, party toxicity scores are much higher than their respective candidates. Figure S4b) shows in-/out-group status cross-sectioned with toxicity scores. In alignment with the literature, out-group mentions are higher than in-group mentions, and this is particularly exacerbated for the KMT. Hou emerges as having the greatest out-group toxicity, likely due to all the comparisons he has to make— not just with Lai and Ko, but also a contender within his own party (as Gou is coded as an out-group for the case of Hou). The low toxicity scores of the DPP and Lai reflect the non-significance of out-group references for Lai.

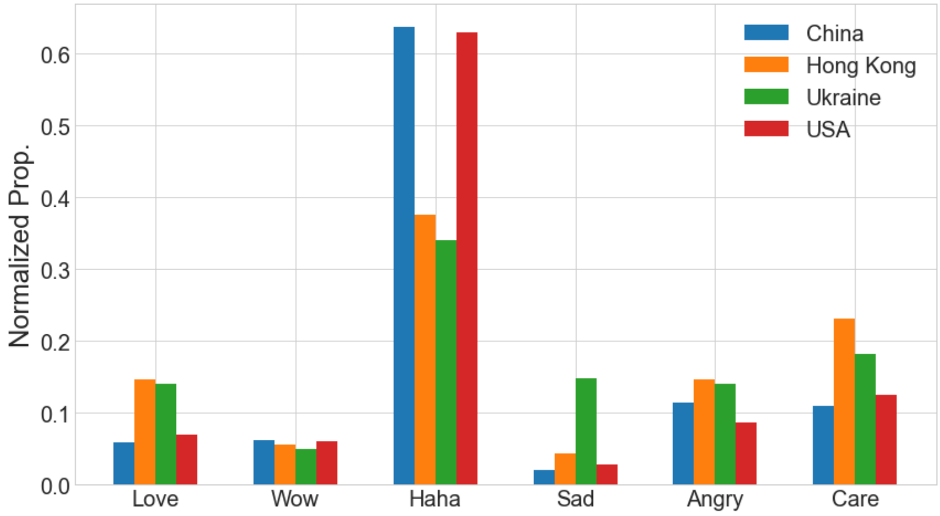

Fig. S3. Geopolitical issues by affective reaction.

Comparison of Keywords			
Topic	Candidate	Keyword List	Love-to-Anger Ratio
USA	Lai	主權、習近平、俄羅斯 (Autonomy, Xi Jing-ping, Russia)	0.709
	Ko	個案、本土、確診 (cases, endemic, confirmed)	0.562
	Hou	台獨、對話、交流、訪美、紐約、華府 (Taiwanese independence, exchange, visit the USA, New York, D.C.)	0.773
	Gou	經濟、發展、科技、利益 (Economics, growth, technology, benefit)	0.722
Technology	Lai	半導體、晶片、AI、台積電、戰爭 (Semi-conductor, chips, AI, TSMC, war)	0.781
	Ko	城市、公園、公宅、台積電、基地 (City, garden, apartment, TSMC, base)	0.700
	Hou	和平、能源、環境、轉型、青年 (Peace, energy, environment, youth)	0.784
	Gou	和平、能源、核電、鴻海、戰爭 (Peace, energy, nuclear energy, FoxConn, war)	0.942

Table S4. List of keywords by topic, with sentiment ratio.

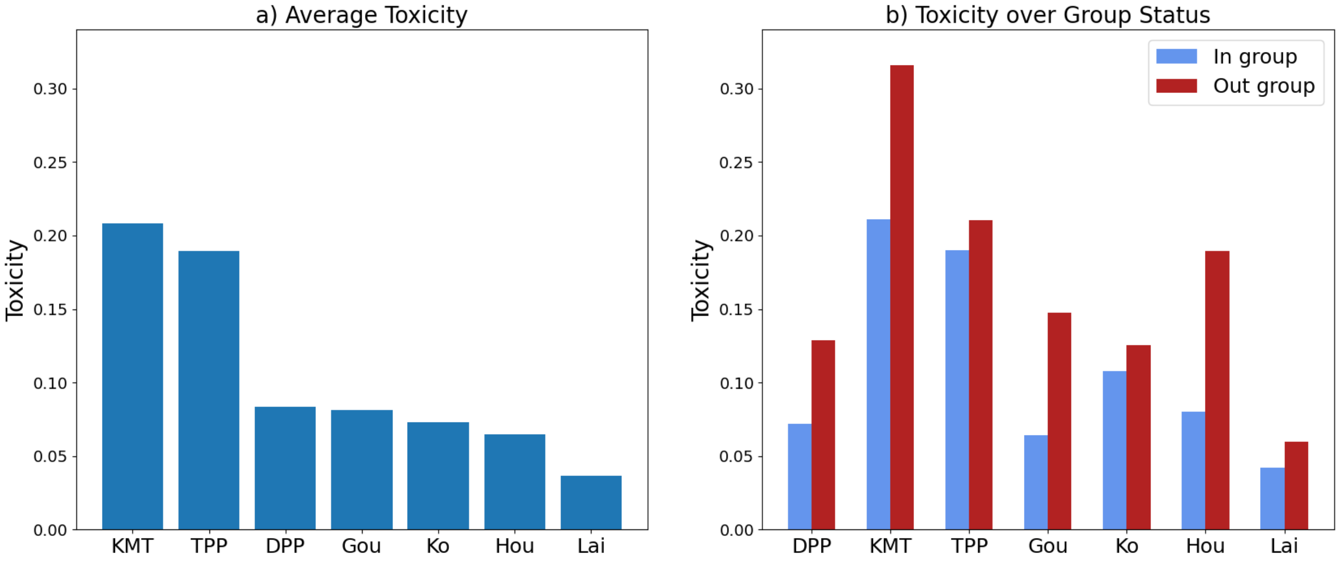

Fig. S4. Average toxicity by candidate for a) overall posts and b) in-group and out-group references.

Public Group Identity	Candidate	Love-Anger Mean
Taiwan	Lai	0.791
Taiwan	Ko	0.353
Taiwan	Gou	0.342
Taiwan	Hou	0.339
R.O.C	Lai	0.428
R.O.C	Ko	0.702
R.O.C	Gou	0.885
R.O.C	Hou	0.746

Table S5. Mean affect of edges between public national identity groups and presidential candidate.

English (translated)	Chinese (original)
Arrogant and Conceited KP	自傲自大的KP
I'm a small vendor who originally wanted to support Ko Wen-je, but now I've changed my mind and support Lai Ching-te because he's more reliable. Because Ko used to oppose the ECFA, but now he wants to restart it in his own political agenda, which is very dangerous.	我是小攤販本來想投柯文哲，如今我改變心意支持賴清德才可靠。因為柯以前反服貿，今在自己國政綱領裡面要重起服貿這樣的人很危險
Lai Ching-te says Taiwan (Republic of China) is already an independent and sovereign country, but both Ko Wen-je and Hou You-yi insist on saying they are "anti-Taiwan independence," meaning they oppose or do not recognize that Taiwan (Republic of China) is already an independent and sovereign country. So, who is the "anti-Taiwan independence" slogan intended for? If it's just to appease the Chinese Communist Party (CCP) out of fear, then why bother running for the President of Taiwan?	賴清德說台灣（中華民國）已是個獨立自主的國家，柯文哲和侯友宜卻異口同聲都說他們「反台獨」，即反對或不承認台灣（中華民國）已是個獨立自主的國家，那麼「反台獨」這個口號是要說給誰聽的呢？ 若因畏懼中共而藉由「反台獨」舔共的話，那又何必左右為難站出來參選台灣總統呢？
Let Terry Gou stay in China, send Ko Wen-je to China, and let Hou You-yi and the Chinese Nationalist Party (KMT) go back to their hometown China. Support Lai Ching-te to protect Taiwan with a high vote count!!	讓郭台銘留在中國把柯文哲送去中國逐侯友宜及中國國民黨滾回老家中國擁賴清德守護台灣##唯一支持賴清德高票當選 ！！
Have you noticed that Lai Ching-te traversed the same journey as us normal Taiwanese people? Quietly doing what he needs to do, not seeking popularity, not causing unnecessary trouble, not engaging in mudslinging, standing firm for democracy, and then letting his opponents - the Communist Party, Xi Jinping, Ko Wen-je, Hou You-yi, Terry Gou... expose themselves. The values of the Taiwanese people are evident, and authoritarian China will eventually decline. God bless Taiwan #TrustTaiwan #TeamTaiwan	有沒有發現賴清德就像我們台灣人一路走來的過程？安靜的做自己該做的事、不嘩眾取寵、不無理取鬧、不謾罵爭辯、堅守崗位挺民主，然後等對手 共產黨/習/柯文哲/侯友宜/郭台銘…自己自爆。台灣人的價值，世人有目共睹，極權中國終會沉淪。天佑台灣 #信賴台灣 #Team台灣
Terry Gou was scolded by a woman while visiting Dongmen Market: "Traitor to Taiwan." She questioned Terry Gou and Hou You-yi for being pro-China and even said Ko Wen-je loves to lie! She applauded with both hands: "The only one who supports Lai Ching-te."	郭台銘逛東門市場一位婦人怒嗆他：「賣台賊」，她質疑郭台銘，侯友宜都親中，還說，柯文哲愛說謊！她雙手在讚：「唯一支持賴清德」。
Hou You-yi can only be a "mayor" of New Taipei City, Terry Gou only wants to be a "district chief" in China's Taiwan region, and Ko Wen-je is only fit to be the "parent" of cross-strait relations. Only Lai Ching-te has the character to be the "President" of Taiwan!!	侯友宜只會選新北市總統級「市長」 郭台銘只想當中國台灣區的「區長」柯文哲只配當兩岸一家親的「家長」 唯賴清德有魄力擔當台灣的「總統」 ！！
Breaking news! Taiwan was almost harmed by Ko Wen-je. In the case of the Twin Stars project in front of Taipei Main Station, the 64.7 billion South Sea Control Stock Corporation had Chinese investment in it. If it hadn't been rejected by the Investment Review Committee and strongly opposed by the Democratic Progressive Party, our national gate would have been opened. Ko Wen-je said that national sovereignty is nonsense. If Taiwan has no national sovereignty, what qualifications does Ko Wen-je have to run for President of Taiwan? What Taiwan people want is to protect Taiwan with Lai Ching-te.	號外！號外！ 台灣差點被柯文哲害死，台北車站前的雙子星案，647億的南海控管股公司，有中資在裡面，當初要不是投審團駁回，跟民進黨極力阻止，我們的國門就被打開柯文哲嗆說國家主權是屁話台灣人民柯文哲既然沒國家主權還有什資格選台灣總統台灣人要選的是守護台灣的賴清德
Vote for Lai Ching-te, and tuition fees will be halved! Vote for Ko Wen-je, and salaries will be reduced to the bone. Don't make the wrong choice! The world was amazed when Lai Ching-te took the exam in the United States and scored 100 points. When the world was surprised by the perfect score, the United States laughed, China got angry, Ko Wen-je panicked, Hou You-yi was dumbfounded, Terry Gou was scared, and the pro-CCP blue and white traitors lost hope.	票投賴清德 學費打對折! 票投柯文哲薪資打到骨折 千萬不要做錯誤選擇! 世界都在看賴清德這次去美國考試 結果竟然考個100分 當世界非常驚訝考試100分時 美國笑了 中國生氣了 柯文哲急了 侯友宜傻眼了 郭台銘嚇尿了 台灣人滿意了 親共藍白賊民死心了
The presidential election poll results are out. Democratic Progressive Party presidential candidate Lai Ching-te is at 35.9%, ranking first, while People First Party presidential candidate Ko Wen-je is at 28.6%, ranking second, and Kuomintang presidential candidate Hou You-yi is at 17.1%, ranking third. However, Wu Zi-jia claims to be a Ko Wen-je supporter (he claims to have spent a lot of money on Ko Wen-je), so it's unclear whether he is influencing the "abandonment effect," meaning that Ko Wen-je's actual support may not be as high, and Hou You-yi's support may not be as low as indicated."	總統大選民調出爐，民進黨總統參選人賴清德35.9%排第一、民眾黨總統參選人柯文哲28.6%排第二、國民黨總統參選人侯友宜17.1%排第三；惟吳子嘉自稱是柯文哲粉絲（自稱為柯文哲花了不少錢），故難說是否在操作棄保效應，即柯文哲實際沒那麼高，侯友宜實際也沒那麼低。

Table S6. 10 samples of references of Ko in Taiwan-based groups.
